# Supplementary figures and images for: BIOME-Preserve: A novel storage and transport medium for preserving anaerobic microbiota samples for culture recovery
Source: PLoS One. 2022 Jan 21;17(1):e0261820. doi: 10.1371/journal.pone.0261820 (PMC8782539; doi:10.1371/journal.pone.0261820)

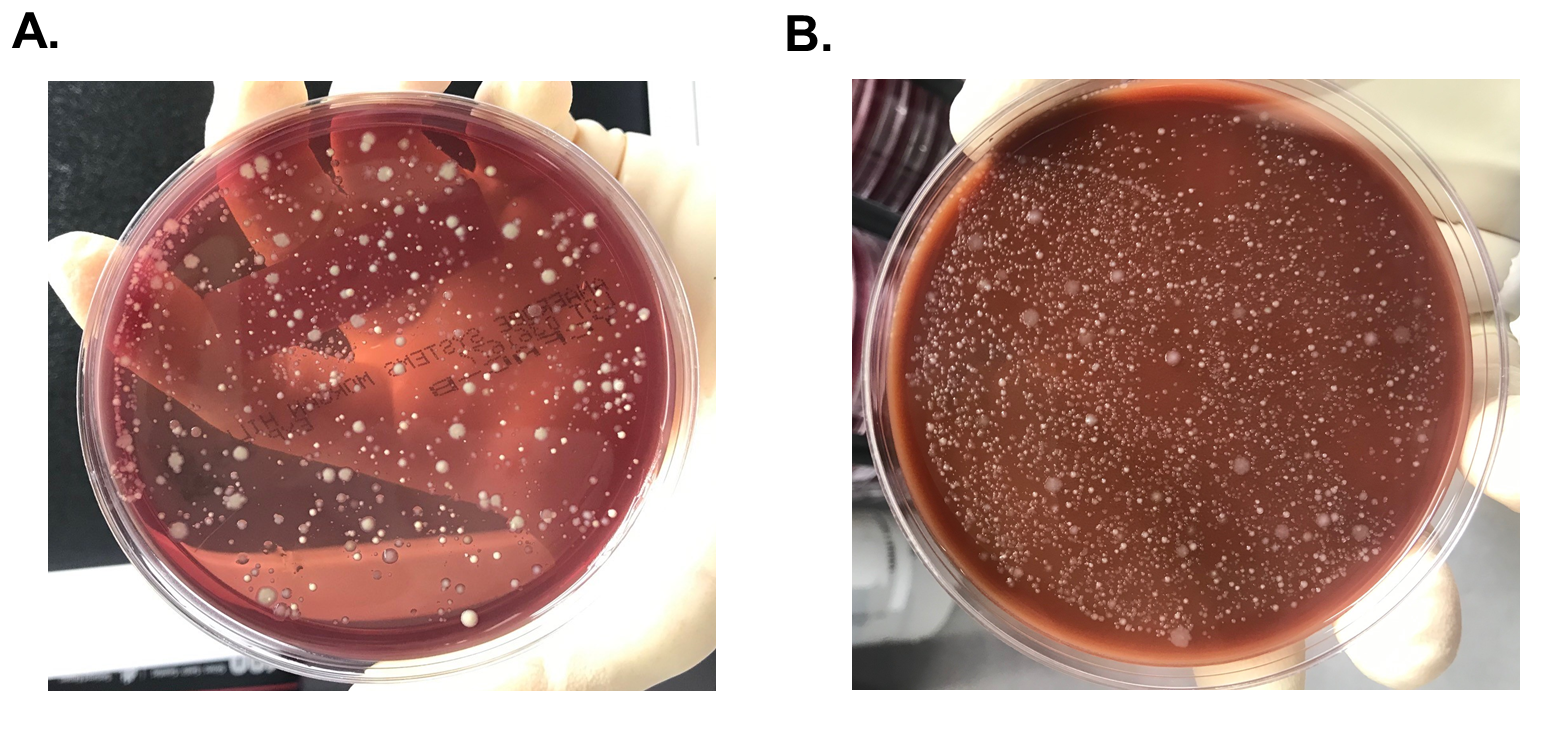

Supplement: S1 Fig — Photographs of representative plates showing culture density on A) plates used to count CFUs (low density) and B) high-density growth plates. (TIF) [file pone.0261820.s001.tif]

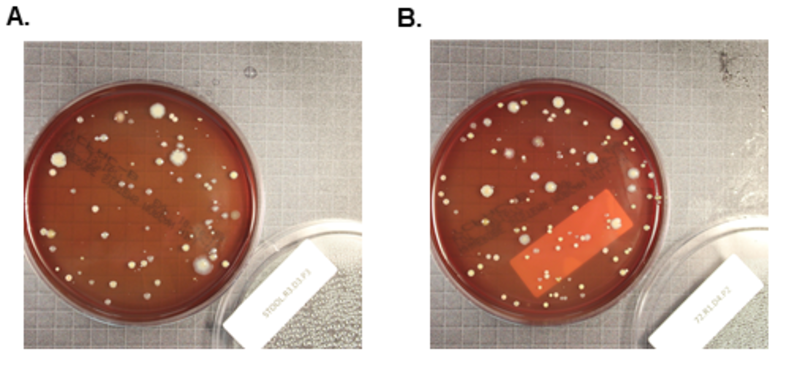

Supplement: S2 Fig — A) 10−8 dilution of fresh stool, B), BIOME-Preserve, 10−6 dilution from fresh stool. This data informed the design of the current study, in we which inoculated the 10−3 dilution from BIOME-Preserve samples onto solid media plates but the 10−5 dilution from fresh stool. (TIF) [file pone.0261820.s002.tif]

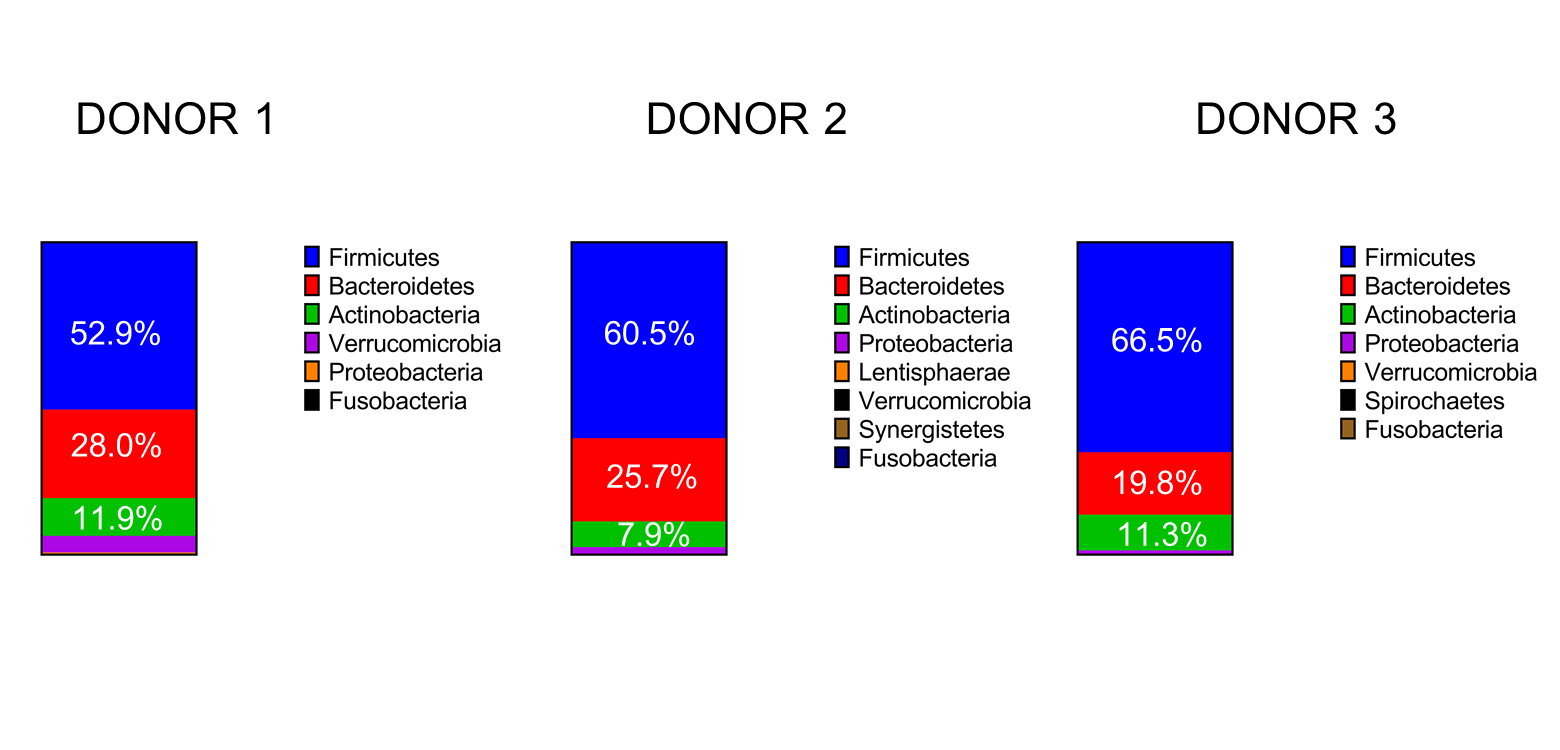

Supplement: S3 Fig — (TIF) [file pone.0261820.s003.tif]
